# Comparative Genomics Analysis of a New *Exiguobacterium* Strain from Salar de Huasco Reveals a Repertoire of Stress-Related Genes and Arsenic Resistance

**DOI:** 10.3389/fmicb.2017.00456

**Published:** 2017-03-21

**Authors:** Juan Castro-Severyn, Francisco Remonsellez, Sandro L. Valenzuela, Cesar Salinas, Jonathan Fortt, Pablo Aguilar, Coral Pardo-Esté, Cristina Dorador, Raquel Quatrini, Franck Molina, Daniel Aguayo, Eduardo Castro-Nallar, Claudia P. Saavedra

**Affiliations:** ^1^Laboratorio de Microbiología Molecular, Departamento de Ciencias Biológicas, Facultad de Ciencias Biológicas, Universidad Andres BelloSantiago, Chile; ^2^Centro de Bioinformática y Biología Integrativa, Facultad de Ciencias Biológicas, Universidad Andrés BelloSantiago, Chile; ^3^Laboratorio de Tecnologías de Membranas, Biotecnología y Medio Ambiente, Departamento de Ingeniería Química, Facultad de Ingeniería y Ciencias Geológicas, Universidad Católica del NorteAntofagasta, Chile; ^4^Laboratorio de Complejidad Microbiana y Ecología Funcional, Instituto Antofagasta and Departamento de Biotecnología, Facultad de Ciencias del Mar y Recursos Biológicos, Universidad de AntofagastaAntofagasta, Chile; ^5^Centre for Biotechnology and BioengineeringAntofagasta, Chile; ^6^Laboratorio de Ecofisiología Microbiana, Fundación Ciencia and VidaSantiago, Chile; ^7^Sys2Diag CNRS/Bio-Rad UMR3145Montpellier, France; ^8^Centro Interdisciplinario de Neurociencia de Valparaíso, Facultad de Ciencias, Universidad de ValparaísoValparaíso, Chile

**Keywords:** *Exiguobacterium*, polyextremophile, stress, comparative genomics, Chilean Altiplano

## Abstract

The Atacama Desert hosts diverse ecosystems including salt flats and shallow Andean lakes. Several heavy metals are found in the Atacama Desert, and microorganisms growing in this environment show varying levels of resistance/tolerance to copper, tellurium, and arsenic, among others. Herein, we report the genome sequence and comparative genomic analysis of a new *Exiguobacterium* strain, sp. SH31, isolated from an altiplanic shallow athalassohaline lake. *Exiguobacterium* sp. SH31 belongs to the phylogenetic Group II and its closest relative is *Exiguobacterium* sp. S17, isolated from the Argentinian Altiplano (95% average nucleotide identity). Strain SH31 encodes a wide repertoire of proteins required for cadmium, copper, mercury, tellurium, chromium, and arsenic resistance. Of the 34 *Exiguobacterium* genomes that were inspected, only isolates SH31 and S17 encode the arsenic efflux pump Acr3. Strain SH31 was able to grow in up to 10 mM arsenite and 100 mM arsenate, indicating that it is arsenic resistant. Further, expression of the *ars* operon and *acr3* was strongly induced in response to both toxics, suggesting that the arsenic efflux pump Acr3 mediates arsenic resistance in *Exiguobacterium* sp. SH31.

## Introduction

Extremophiles are microorganisms from all three domains of life (Bacteria, Archaea and Eukarya) that grow in the most hostile environments found on Earth, where they must withstand conditions including extreme pH, temperature, salinity, pressure, UV radiation, and the presence of heavy metals or toxic compounds. In some cases, extremophiles face more than one extreme condition simultaneously, and are called polyextremophiles (Seufferheld et al., 2008; Bowers et al., 2009; Farías et al., 2013; Kurth et al., 2015). To survive under extreme conditions, microbes finely tune gene expression, modulating the levels of proteins implicated in the response to stress (Duché et al., 2002). Since their discovery, extremophiles have attracted researchers because of their unique physiology, ability to adapt to different environments, and for their potential use in biotechnology (Rothschild and Mancinelli, 2001).

The Andean Plateau region in West Central South America, the Altiplano, hosts a rich microbial diversity and remains an untapped resource for understanding the genetic diversity and distribution of polyextremophiles (Catalán et al., 2006). High-altitude Andean Lakes (HAALs) are exposed to some of the highest levels of solar radiation on Earth (Albarracín et al., 2015). Further, water bodies found in the Altiplano (i.e., shallow lakes, ponds, streams, etc.) are highly diverse, showing different chemical compositions, temperatures, evaporation rates, and depths, among other conditions. Together, these factors drive the changing community structure of rich polyextreme microbiota that populates these lakes (Márquez-García et al., 2009; Dorador et al., 2013; Cordero et al., 2014). Despite these harsh conditions, almost 500 strains of prokaryotes (archaea, cyanobacteria and eubacteria) and lower eukaryotes (fungi and yeast) have been isolated from bacterioplankton, benthos, microbial mats, and soils surrounding HAALs (Dib et al., 2008; Flores et al., 2009; Ordoñez et al., 2009; Di Capua et al., 2011; Lynch et al., 2012; Bull and Asenjo, 2013; Bull et al., 2016). Microbial communities that evolved in HAALs tolerate a wide range of chemical and physical stresses, including wide fluctuations in temperature, low nutrient levels, alkalinity, and hypersalinity (up to 30%). Further, high concentrations of heavy metals and metalloids, especially arsenic (up to 200 ppm), are found in HAALs, primarily because of the geological foundation (Fernandez-Zenoff et al., 2006; Zenoff et al., 2006; Dib et al., 2009; Farias et al., 2009; Albarracín et al., 2012).

Salar de Huasco is a saline wetland located at 3,800 m above sea level in the Chilean Altiplano (-20.303436, -68.880918) and is considered an athalassohaline system. It exhibits typical conditions of the Altiplano: low temperatures (mean annual temperature <5°C), low atmospheric pressure, high solar radiation (up to 72 Wm^-2^ UVA radiation: Hernández et al., 2016), negative water balance, and variable salinity ranging from freshwater to saturated salt waters. Despite these conditions, communities composed of microbial groups from all major branches of the microbial tree of life, including *Proteobacteria, Cyanobacteria, Bacteroidetes*, and *Archaea*, thrive and grow in water bodies of the Salar de Huasco (Dorador et al., 2008a,b, 2009, 2010, 2013; Aguilar et al., 2016; Molina et al., 2016). It has been proposed that this exceptional microbial diversity results from species diversification, caused by the adaptation of microorganisms to the constantly changing environment (Albarracín et al., 2016). Consequently, Salar de Huasco represents a potential reservoir of undescribed microbial diversity that could serve to study and identify novel mechanisms of stress resistance.

The *Exiguobacterium* genus (Collins et al., 1983) includes psychrotrophic, mesophilic, and moderate thermophilic species with variable morphologies (ovoid, rods, double rods, and chains) that grow in a wide range of habitats, including cold and hot environments with temperatures ranging from -12 to 55°C (Vishnivetskaya and Kathariou, 2005; Vishnivetskaya et al., 2007). Taxonomic and phylogenetic classification of the genus by 16S rRNA gene sequence analysis shows that *Exiguobacterium* forms two distinct groups: Group I includes strains isolated from cold or low temperature environments, and Group II from hot springs or slightly alkaline and marine environments (Vishnivetskaya et al., 2009). One exception is *Exiguobacterium* sp. GIC31, which belongs to group II, but was isolated from a glacier in Greenland (Vishnivetskaya et al., 2014).

Members of the *Exiguobacterium* genus display traits that could be of biotechnological interest, e.g., bioremediation and agriculture applications. For example, strains Z8 and 2Sz can neutralize highly alkaline industry wastewater and remove pesticides, respectively (Lopez et al., 2005; Kumar et al., 2006); strain WK6 reduces arsenate to arsenite (Anderson and Cook, 2004), and other strains reduce chrome and mercury in a wide range of temperatures, pH, and salt concentrations (Pattanapipitpaisal et al., 2002; Petrova et al., 2002; Okeke, 2008); *Exiguobacterium oxidotolerans* T-2-2T shows high catalase activity in response to H_2_O_2_ as a function of the growth phase and oxygen levels, which could be used to remove peroxides used in the bleaching industry (Takebe et al., 2007); *Exiguobacterium* sp. S17, isolated from Laguna Socompa in the Argentinian altiplanic desert, tolerates high arsenic concentrations (Belfiore et al., 2013; Ordoñez et al., 2013). Further, members of the *Exiguobacterium* genus are a source of enzymes that exhibit a broad range of thermal stability (Usuda et al., 1998; Suga and Koyama, 2000; Hwang et al., 2005; Hara et al., 2007; Kasana and Yadav, 2007).

In the present study, we aimed to understand the phylogenetic placement and to identify genetic determinants required for the response to stress in a new psychro-halotolerant strain isolated from Salar de Huasco, *Exiguobacterium* sp. SH31. We investigated whether *Exiguobacterium* sp. SH31 belongs to Group II, and to what extent species from this genus share functional features based on the group to which they belong. Additionally, we searched the genomes of *Exiguobacterium* sp. SH31 and other species for the presence of genes encoding proteins known to be involved in stress and metal resistance, e.g., arsenic, copper, cadmium, and oxidative and UV stress. Finally, we show that *Exiguobacterium* SH31 is resistant to arsenic, which might be mediated, at least in part, by the Acr3 arsenic efflux pump.

## Materials and Methods

### Bacterial Growth Conditions and DNA Extraction

*Exiguobacterium* sp. SH31 was isolated from Salar de Huasco, an altiplanic shallow saline wetland in the Atacama Desert, Chile, located between -20.303436, -68.880918 at an altitude of 3,800 m. Salinity of the sampling point was 12.3% NaCl, with 20.6 mS/cm of conductivity, pH 8.8 and 8°C at the sampling point. Strain SH31 was grown for 48 h at room temperature (∼25°C) with shaking at 120 rpm in 1 L of YP culture media (2 g/L yeast extract, 5 g/L Peptone and 25 g/L NaCl), with a 10% v/v inoculum. DNA extraction was performed with the AxyPrep Bacterial Genomic DNA miniprep kit (AXYGEN) following the manufacturer’s instructions. DNA quantity and quality was determined spectrophotometrically (OD_260/280_ ratio).

### DNA Hybridization Assay

To assess the relatedness of *Exiguobacterium* SH31 to the type strain *Exiguobacterium aurantiacum* DSM 6208^T^, we performed a hybridization assay in 2 X SSC + 5% formamide medium at 69°C. We evaluated the results per the recommendations of the *ad hoc* committee for the definition of bacterial species (threshold value of 70% DNA–DNA similarity; Wayne et al., 1987). The assay was performed at the Leibniz Institute DSMZ – German Collection of Microorganisms and Cell Cultures^1^.

### Sequencing and Assembly

Purified genomic DNA was used to construct two different types of sequencing libraries, namely a single-end (prepared from 500 ng of fragmented DNA (nebulization)) and a mate paired-end (prepared with 5 μg of DNA fragmented by hydroshear), using the GS FLX Titanium Rapid Library, GS FLX Titanium Rapid Library prep Paired-End Adaptors, and FastStart PCR System kits, following the manufacturer’s recommendations (average fragments size was between 500 and 900 bp). An equimolar mix was then sequenced using the Roche 454 FLX platform. SFF files were separated into two FASTQ files, one for each library type, using sff_extract utility from seq_crumbs^2^. Single-end reads were trimmed at <Q17 and all the resulting reads of <70 bp were discarded. Reads longer than the targeted length (500 bp) were clipped at the 3′ end so that only reads <500 bp were used. Mate paired-end reads were separated into two groups, corresponding to each sequenced end of the fragment (5′ and 3′ ends reads of the sequenced fragment) and both groups were saved as backward sense reads. Trimming was performed as described for single-end reads. Only those paired reads that conserved their mates were employed for the assembly. The final number of reads obtained after this procedure was 81.675 single-end and 61.226 mate paired-reads (approximately 11X and 4X depth coverage respectively), with a read length average of 200 bp after trimming internal adapters. Assembly was performed using both mate paired and single-end reads as in the Celera genome assembler version 7.0 (Myers et al., 2000); unitigger BOG, unitigger error ratio 0.03, with the final parameter values by default. The final assembly included 120 contigs with a length of 3,031,801 bp. The Whole Genome Shotgun project has been deposited at DDBJ/ENA/GenBank under the accession LYTG00000000. The version described in this paper is LYTG01000000 (Bioproject PRJNA319980).

### *Exiguobacterium* Genomic Dataset

All available genome sequences used in the analyses have been deposited in GenBank as of December 2015 (Accession numbers in Supplementary Table S1), including a status of complete, scaffolds, or contigs. The resulting 33 genomes were organized into 18 described species plus 15 not classified at the species level. All the genome sequences (including the new *Exiguobacterium* sp. Strain SH31 from this study), were re-annotated using a combination of *ab initio* and similarity methods as implemented in Prokka version 1.10 (Seemann, 2014) and NCBI Prokaryotic Genome Annotation Pipeline (Tatusova et al., 2016). Some genomes were also annotated using the following databases: Gene3d 3.5.0, HAMAP 201511.02, PANTHER 10.0, PFam 28.0, PIRSF 3.01, PRINTS 42.0, ProDom 2006.1, prosite 20.113, SignalP 4.0, Smart 6.2, SuperFamily 1.75, TigerFam 15.0, and TMHMM 2.0, as implemented in InterProScan.5.16-55.0 (Jones et al., 2014; Mitchell et al., 2014).

### Circular sp. SH31 Genome Map Generation

The plotMyGBK^3^ pipeline was used to generate a circular map of the *Exiguobacterium sp.* SH31genome. The plotMyGBK is written in Python and extracts contigs from a.gbk file using the SeqlO module generating a.faa file that is then used to search for genes on the conserved domains database (CDD) through RPS-blast using an *e-value* of 1E^-2^ to avoid false positives. Then, genes were classified according to the Clusters of Orthologous Groups (COG) nomenclature (Marchler-Bauer et al., 2003). Finally, COG annotations were used to plot genes, GC content, GC skew, tRNA and rRNA positions, using the OmicCircos v 1.8.1. R package^4^ (Hu et al., 2015).

### Orthologous Gene Search

Bi-directional best hit searches using Blast was used to infer homology between reference genes from Swiss-prot and the predicted genes from our *Exiguobacterium* dataset (Altschul et al., 1990). A minimum *e-value* of 1E^-05^ and a query coverage filter of 85% was used to avoid partial alignments. This strategy was used to compare stress-response genes and genes involved in metal/metalloid resistance/tolerance.

### Phylogenetic Analysis

Thirty phylogenetic gene markers implemented in AMPHORA2 were used (*dnaG, frr, infC, nusA, pgk, pyrG, rplA, rplB, rplC, rplD, rplE, rplF, rplK, rplL, rplN, rplP, rplS, rplT, rpmA, rpoB, rpsB, rpsC, rpsE, rpsI, rpsJ, rpsK, rpsM, rpsS, smpB*, and *tsf)* from the data set (Wu and Scott, 2012). All nucleotide sequences were translation aligned using MAFFT (Katoh and Standley, 2013) as implemented in Geneious v 7.1.9 software (Kearse et al., 2012). The alignments were then concatenated using Seqotron version 1.0.1 (Fourment and Holmes, 2016). A distribution of probable trees using Bayesian Inference as implemented in MrBayes 3.2.5 was used. Two separate runs of 20 million generations were executed (four chains each run; sampling every 1000 generations; nst = 6, nucmodel = 4by4, rates = gamma ngammacat = 4). Then, the tree distributions were summarized as the Maximum a Posteriori estimate removing the first 5 thousand trees as implemented in SumTrees from DendroPy 4.1.0 (Sukumaran and Holder, 2010). The phylogeny and stress-response gene matrix was plotted using the R package ggtree v 1.0.21 (Yu et al., 2016).

### Average Nucleotide Identity Analysis

Genome-wide nucleotide identity trends were explored in the genome dataset by estimating all-against-all pairwise Average Nucleotide Identity (ANI). The ANIm approach that uses MUMmer (NUCmer) was used to align the input sequences as implemented in pyANI (Kurtz et al., 2004^5^). The average between any given pair was used as the final value. Heat maps were generated using pheatmap V 1.0.8 R package (Kolde, 2015). Genomic clusters were defined using hierarchical clustering (method: average) based on a 75% ANI threshold, which represents a conservative boundary for genus/species level classification (Qin et al., 2014).

### Pan Genome Analysis

Protein-coding gene families were defined as gene clusters with at least 70% sequence identity using three different methods (COG triangle, orthoMCL and bidirectional best-hit) as implemented in GET_HOMOLOGUES v 2.0 (Contreras-Moreira and Vinuesa, 2013). The core genome was defined as the set of genes present in at least 95% of the genomes. Likewise, the accessory genome set was defined as genes present in less than 95% of genomes. This analysis was performed at two levels: the complete 34 genome dataset, and on each of the ANI 75% clusters. To gain insights into the functional potential of the accessory genome, the core genome was subtracted from each of the 34 genomes and annotated the remaining genes using Gene Ontology (GO) terms (Ashburner et al., 2000). All plots were generated in the ggplot2 R package (Wickham, 2009).

### Functional Analysis of the Core and Accessory Genomes

The core and accessory genomes were separated for each of the six 75% ANI clusters, and GO terms were assigned to both sets. Gene locus tags were used to map GO terms from the Pfam entries (supplied by InterProScan5 annotated file) for each genome. GO terms for each gene cluster was converted to their parent GO terms at the second level on a GO graph (go-basic.obo obtained from http://geneontology.org/page/download-ontology) (The Gene Ontology Consortium, 2015). Statistically significant functional terms between core and accessory genomes were determined by applying the Mann–Whitney–Wilcoxon test (*p-value* <0.05). Results were also plotted with the ggplot2 R package (Wickham, 2009).

### Growth Curves, Minimum Inhibitory Concentration (MIC), and Expression of Arsenic Resistance Genes

Growth curve assays were performed to determine the ability of *Exiguobacterium* SH31 to grow at different concentrations of arsenite (AsIII) and arsenate (AsV). Briefly, a liquid culture of *Exiguobacterium* SH31 grown to an OD_600_ = 0.4 (LB medium) was diluted by adding liquid media supplemented with different concentrations of arsenic species: AsIII (NaAsO_2_; 4, 6, 10 mM) and AsV (Na_2_HAsO_4_; 25, 50, 100 mM) until reaching an OD_600_ ≈ 0.004. Growth (25°C) was monitored in a microplate reader (TECAN Infinite 200 PRO Nanoquant) at 600 nm every 60 min over a 48-h period. MIC was determined from a bacterial culture grown in LB medium (OD_600_ ≈ 0.004) by taking a 190 μL aliquot and diluting it with solutions of AsIII and AsV to final concentrations of 1–15 mM and 5–500 mM, respectively (200 μL final volume). Then, samples were incubated at 25°C for 72 h with constant agitation, and the OD_600_ was measured in a TECAN Infinite 200 PRO Nanoquant.

Finally, to determine the relative expression of genes known to be involved in arsenic resistance and present in *Exiguobacterium* SH31, the transcript levels of *arsB, arsR, arsC* and *acr3*, were quantified by qRT-PCR. *Exiguobacterium* SH31 was grown in LB medium at 25°C with constant agitation until reaching an OD_600_ of 0.4. The culture was divided into three fractions: control, 3 mM NaAsO_2_, and 20 mM Na_2_HAsO_4_, and incubated for 20 min. RNA extraction was carried out using the Phenol/Chloroform method and RNA integrity was monitored using 1% agarose electrophoresis. cDNA was synthesized using the M-MLV Reverse Transcriptase kit (Promega) and Random Primer oligonucleotides hexamers (Invitrogen^TM^). The PCR reaction was carried out as follows: 10 min at 95°C followed by 40 amplification cycles (95°C × 30 s, 58°C × 30 s; 72°C × 30 s), and a final step of 95°C × 15 s; 25°C × 1 s; 70°C × 15 s and 95°C × 1 s) using primers specific for each gene (Primers in Supplementary Table S2). Transcript levels were quantified using the Brilliant II SYBR Green QPCR Master mix kit (Agilent Technologies) on a Stratagene Mx3000P thermal cycler. Relative expression was normalized using 16S rRNA gene expression levels (Pfaffl, 2001).

### Modeling and Alignment of ACR3

Structural modeling and visualization of Acr3 from strains SH31 and S17 strains was performed using iterative threading assembly refinement (I-TASSER software) (Yang et al., 2015).

## Results

Strain SH31 showed a 99% similarity at the 16S rRNA gene sequence with *Exiguobacterium aurantiacum* DSM 6208 (type strain) and *Exiguobacterium* sp. S17 (Argentinian Altiplano), and was classified as a member of the *Exiguobacterium* genus. However, an *in vitro* DNA–DNA hybridization assay revealed that SH31 does not belong to the same species than *E. aurantiacum* DSM 6208 (39.5% similarity; threshold is 70% for species; Wayne et al., 1987; Goris et al., 2007).

### Genome Assembly and Nucleotide Identity Analysis

**FIGURE 1:**
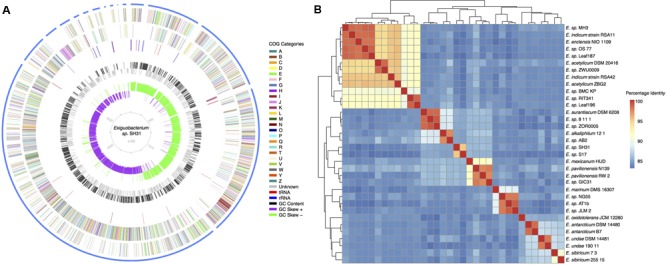
**(A)** Circular map of the *Exiguobacterium sp*. SH31 draft genome (3031801 bases, 3214 genes, 23 rRNA and 51 tRNA). Gene functions were annotated based on COG categories: (A) RNA processing and modification 0.01%; (B) Chromatin structure and dynamics 0.03%; (C) Energy production and conversion 4.5%; (D) Cell cycle control, cell division, chromosome partitioning 1.9%; (E) Amino acid transport and metabolism 10.3%; (F) Nucleotide transport and metabolism 1.6%; (G) Carbohydrate transport and metabolism 5.6%; (H) Coenzyme transport and metabolism 3.6%; (I) Lipid transport and metabolism 1.4%; (J) Translation, ribosomal structure and biogenesis 3.3%; (K) Transcription 4.8%; (L) Replication, recombination and repair 3.9%; (M) Cell wall/membrane/envelope biogenesis 4.3%; (N) Cell motility 1.3%; (O) Posttranslational modification, protein turnover, chaperones 4.4%; (P) Inorganic ion transport and metabolism 12.6%; (Q) Secondary metabolites biosynthesis, transport and catabolism 2.5%; (R) General function prediction only 19.3%; (S) Unknown function 3.4%; (T) Signal transduction mechanisms 7.1%; (U) Intracellular trafficking, secretion, and vesicular transport 0.9%; (V) Defense mechanisms 3.3%; (W) Extracellular structures 0.0%; (Y) Nuclear structure 0.0%; (Z) Cytoskeleton 0.02%. **(B)** Average Nucleotide Identity of 34 *Exiguobacterium* isolates. *Exiguobacterium* sp. SH31 is very distinct and is most similar to isolate *Exiguobacterium* S17.

*Exiguobacterium* genomes range from 2.8 to 3.3 Mb in length with an average GC content of 46–53%. The genome of *Exiguobacterium* sp. strain SH31 resulted in 120 contigs totaling 3.03 Mb in length with a GC content of 51.6%. In agreement with what was found in other isolates, 2992 coding sequences (CDS; including 902 hypothetical proteins), 23 ribosomal RNA (rRNA) genes, and 49 transference RNA (tRNA) genes were identified in strain SH31. Most of the CDS found in *Exiguobacterium* sp. strain SH31 were annotated using the COG system (2,079 genes annotated; **Figure 1A**).

To understand the overall genetic similarity of strain SH31 with members of the *Exiguobacterium* genus, an ANI analysis was conducted (Konstantinidis and Tiedje, 2005a,b; Goris et al., 2007; Richter and Rosselló-Mora, 2009) against other 33 publicly available *Exiguobacterium* genomes (Supplementary Table S1). The analysis revealed distinct genomic clusters and that strain S17 was the most similar isolate to SH31 with a 95% ANI (**Figure 1B**), suggesting that they belong to the same species, according to the classification defined by Goris et al. (2007). Strain S17 was isolated from a stromatolite sample of Lake Socompa in the Argentinian Altiplano (-24.525681, -68.207912), an environment with similar conditions to Salar de Huasco (Cabrol et al., 2007; Ordoñez et al., 2009; Albarracín et al., 2015). In general, as previously reported (Vishnivetskaya et al., 2009), isolates tended to cluster by habitat, i.e., strains isolated from cold environments were more similar to each other than to strains isolated from marine/alkaline/temperate environments, and vice versa. Using the proposed boundaries for species delimitation, i.e., 95% ANI (Varghese et al., 2015), 21 clusters were recognized. Because at a 95% ANI clusters were mostly composed of singletons, we used a more conservative estimate for species/genus boundary. Using a 75% ANI cut-off value, six clusters were obtained: **cluster I** (12 isolates) *E.* strain MH3, *E. indicum* RSA11, *E. enclensis* NIO-1103, *E.* strain OS-77, *E.* strain leaf 187, *E. acetylicum* DSM 20416, *E.* strain ZWU0009, *E. indicum* RSA42, *E. acetylicum* ZBG2, *E.* strain BCM-KP, *E.* strain RIT341, and *E.* strain leaf 196; **cluster II** (4 isolates) *E. marinum* DSM 16307, *E.* strain NG55, *E.* strain AT1b, and *E.* strain JLM-2; **cluster III** (7 isolates) *E. oxidotolerans* JCM 12280, *E. antarcticum* DSM 14480, *E. antarcticum* B7, *E. undae* DSM 14481, *E. undae* 190-11, *E. sibiricum* 7-3, and *E. sibiricum* 255-15; **cluster IV** (5 isolates) *E. aurantiacum* DSM 6208, *E.* strain 8-11-1, *E.* strain ZOR0005, *E. alkaliphilum* 12/1, and *E.* strain AB2; **cluster V** (2 isolates) *E.* strain SH31 and *E.* strain S17; **cluster VI** (4 isolates) *E. mexicanum* HUD, *E. pavilionensis* N139, *E. pavilionensis* RW-2, and *E.* strain GIC31.

Because *Exiguobacterium* sp. SH31 thrives in an environment where it is exposed to multiple stress conditions including UV radiation, high salinity, and high temperature, among others, we hypothesized that its genome encoded known genetic determinants required for stress resistance/tolerance. Using BLAST searches against SwissProt protein sets for known stress related-genes, we found that strain SH31 possesses several genes encoding proteins related to the response or adaptation to oxidative stress detoxification (*katA, katE, sodA, gpo, ahpC, ahpF, ohrR*); DNA repair systems (*uvrABC, mutLS, mutM, ruvAB, rec* systems, *ssb, lexA*); photolyases (*phrB, cry*-DASH); photoprotective pigments (*car, crt* systems); osmotic stress (*opu* systems, *putP, gltA, glnA, proC*); heat shock (*dnaJK, groLS, htpG, hrcA*); cold shock (*csp*); capsule biosynthesis (*capAD, pgaB, pgaC*); and resistance to toxic compounds such as antibiotics (*marR, liaRS*) and heavy metals (arsenic *arsA, arsB*; cadmium *cadA, cadC*; copper *copA, csoR*; mercury *merA, merB, merR*; tellurium *tehB, terC*; chromium *chrR, srpC*). In agreement with the ANI analysis, strain SH31 shared many of these determinants with its closest relative, strain S17, including the *acr3* gene that encodes an accessory or secondary arsenic efflux pump (see Ordoñez et al., 2015).

### Phylogenetic Analysis of *Exiguobacterium* sp. Strain SH31

**FIGURE 2:**
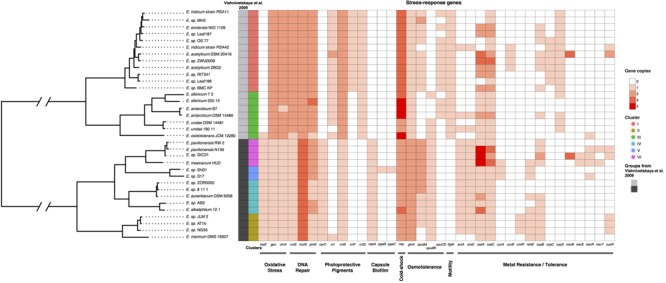
**Multi-locus phylogenetic analysis of 34 *Exiguobacterium* isolates.** Tree: Mid-point rooted phylogeny of *Exiguobacterium* recovers Group I and II phylogenetic groups. Note that *Exiguobacterium* sp. SH31 is part of Group II. *Matrix*: Distribution and copy number of stress-related genes among *Exiguobacterium* genomes. The first two columns indicate Vishnivetskaya et al., 2009 groupings and 75% ANI clustering, respectively. Stress-related genes are color-coded by copy number.

We further investigated if members of the *Exiguobacterium* genus showed a similar pattern as in the ANI analysis, but under a phylogenetic framework. To avoid the detrimental effects of horizontally transferred genes in phylogenetic inference, 30 genes related to ribosome and replication were selected to infer the phylogenetic relationships of the group (Bayesian Inference, partitioned analysis; see Materials and Methods). Interestingly, the topology of the tree mirrors the classification proposed by Vishnivetskaya et al. (2009) and is consistent with the ANI results (**Figure 2**; column 1 and 2). Some species seem to be monophyletic (*E. undae; E. antarcticum; E. sibiricum*), while others do not form monophyletic groups (*E. indicum; E. acetylicum*), suggesting that more extensive sampling and clearer taxonomic classification schemes are needed.

A previous work investigated the genomes of several *Exiguobacterium* strains to determine if there could be group-specific genes by using southernblot hybridization and *Exiguobacterium sibiricum* 255-15 as the reference strain. Group II does not contain the selected genes or may contain them with <70% identity (Vishnivetskaya et al., 2009). Given the current availability of genome sequences, we set out to confirm if the group pattern found by Vishnivetskaya et al. (2009) was also present in our dataset. Using a bidirectional blast hit approach, 15 of the 16 genes described by Vishnivetskaya et al. (2009) were found to be present in both groups. This finding was confirmed by annotating those genes using the InterProScan5 suite, with similar results. The only gene found exclusively in one group was *flgM* (**Table 1**).

**Table 1:** Genes used as conservative markers of the two distinctive *Exiguobacterium* groups by Vishnivetskaya et al. (2009).

Function	Gene ID^∗^	Genes	Detectable homologous			
			Vishnivetskaya et al., 2009		This work	
			Group I	Group II	Group I	Group II
Replication and repair	Exig_0006	*gyrA*	+	-	+	+
	Exig_0005	*gyrB*	+	+	+	+
	Exig_2932	*srmB/csh*	+	-	+	+
	Exig_3033	*dnaB*	+	-	+	+
Transcription	Exig_2463	*flgM*	+	-	+	-
	Exig_0137	Anti-sigma factor	+	-	+	+
	Exig_0136	*rpoE*	+	-	+	+
Translation and biogenesis	Exig_0119	*infA*	+	+	+	+
	Exig_1837	*infB*	+	+	+	+
	Exig_0047	RNA-binding S4	+	-	+	+
	Exig_2427	Sigma54 S30EA	+	+	+	+
Posttranslational modification,	Exig_2147	*tig*	+	-	+	+
chaperones	Exig_0781	*dnaK*	+	+	+	+
	Exig_2768	*groL*	+	+	+	+
Signal transduction	Exig_0364	*uspA*	+	-	+	+
Lipid metabolism	Exig_2596	*desA*	+	+	+	+

*^∗^Gene IDs are given as defined in http://genome.ornl.gov/microbial/exig/.*

Using the same bidirectional BLAST-based approach and validation with InterProScan5, the genomes of all 34 *Exiguobacterium* strains were searched for the presence of stress-response genes. The analysis showed that there are differences between the two groups (**Figure 2**; matrix), which were observed in *katE, ohrA* and *gpo* (oxidative stress compound detoxification), *carD* and *crtB* (pigment biosynthesis), *capA* (capsule biosynthesis), *glnA* (osmoadaptation), *csp* (cold shock), *mutS* and *ruvB* (DNA repair) genes. The copy number of stress-response genes correlated with the environmental conditions from where each strain was isolated, e.g., *E. oxydotolerans* genome encodes proteins required for the response to oxidative stress and *E. antarcticum* and *E. sibiricum* for cold shock. A full list of stress-response genes analyzed and their copy numbers is found in Supplementary Table S3.

### Pan Genome and Gene Ontology Analysis

As the strains used for the analyses come from disparate environments (Supplementary Table S1), we evaluated whether the core and accessory genome varied in size and biological function (by GO). *Exiguobacterium* genomes are highly divergent (**Figure 1B**), and hence, a pan genome analysis was conducted (at 70% identity; pan genome size = 7716). The analysis showed that the core genome is composed of 1,798 genes (23.3%) and the accessory genome of 5,918 genes (76.7%). The pan genome size and functional composition was also evaluated according to clusters defined at 75% ANI. Overall, pan genome size ranged from 22 to 50%, was consistent between groups, and increased according to the number of genomes within a cluster (**Table 2**). Cluster V, which only includes the genomes of strains S17 and SH31, had the largest accessory genome sampled so far in the genus, explaining the increased genome size of strains in this cluster. These results highlight the high degree of variability in gene content among strains from the *Exiguobacterium* genus, and suggest that a more thorough sampling of the Altiplano region would likely yield novel gene families.

**Table 2:** Pan genome results of the six *Exiguobacterium* strains clusters.

Cluster	# Strains	Pan genome	Core genome^∗^	Accessory genome^∗^
I	12	4684	2722 (58,1%)	1962 (41,9%)
II	4	3557	2754 (77,4%)	803 (22,6%)
III	7	4310	2615 (60,7%)	1695 (39,3%)
IV	5	3649	2604 (71,4%)	1045 (28,6%)
V	2	3632	2466 (67,8%)	1166 (32,2%)
VI	4	3658	2814 (76,9%)	844 (23,1%)

*Asterisks indicate the number of gene families (percentage of core or accessory genes on the pan genome of each cluster).*

To further investigate the composition of the accessory genome, we interrogated gene distribution according to GO categories. Accessory genes related to the GO categories Binding and Catalytic Activity were more abundant within the accessory genome, while other functions such as Antioxidant Activity, Biological Regulation, and Cell Part were homogeneously distributed (Supplementary Figure S1A). Interestingly, the genomes of cluster V (strains SH31 and S17) showed a higher proportion of genes belonging to GO categories such as Catalytic activity, Cell part, Membrane, Metabolic process, Structural molecule activity, and Cellular process (Supplementary Figure S1B).

### Metal/Metalloid Response Genes in *Exiguobacterium* Genomes

**FIGURE 3:**
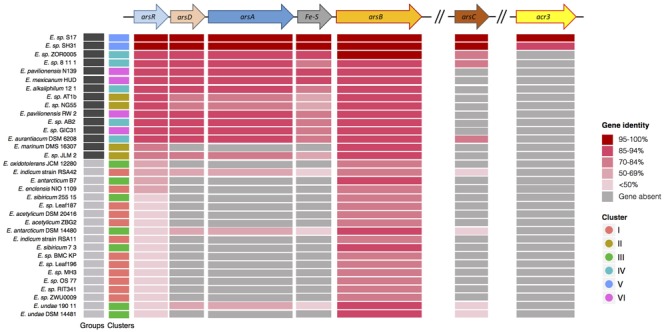
**Arsenic resistance genes in sequenced *Exiguobacterium* strains Genetic organization of the genes colored by function.** *arsR* arsenical resistance operon repressor; *arsD* arsenical resistance operon *trans*-acting repressor; *arsA* arsenical pump-driving ATPase; Fe–S putative iron sulfur protein; *arsB* arsenical pump membrane protein; *arsC* arsenate reductase; *acr3* arsenite efflux pump. Heat scale shows identity percentage of all strains sequence, using as reference the *Exiguobacterium* S17 genes. The first two columns indicate Vishnivetskaya et al. (2009) groupings and 75% ANI clustering, respectively.

The genomes of all 34 strains were searched for the presence and patterns of metal/metalloid response genes, and whether their distribution matched the phylogenetic and ANI classifications. The analysis revealed the presence of several genes involved in metal/metalloid response, of which most were related to cobalt, zinc, cadmium, copper, mercury, tellurium, chromium, and arsenic resistance (Supplementary Table S4). Previous studies showed that strain S17 is resistant to arsenic (Belfiore et al., 2013; Ordoñez et al., 2015) and that it encodes a secondary arsenic efflux pump, named Acr3. Because SH31 and S17 are closely related, we investigated if strain SH31 and other members of the *Exiguobacterium* genus also encoded the genetic determinants required for arsenic resistance, including *acr3*. For this purpose, strain S17 was used as a reference. Based on the presence and identity of the genes and operon organization, two distinct patterns were observed. In most cases, these patterns matched the two phylogenetic groups described before, with the exception of *E. marinum* DSM 16307 from group II and *E. indicum* RSA42, *E. antarcticum* DSM 14480, and *E. undae* 190-11 from group I (**Figure 3**). The *acr3* (arsenite efflux pump) gene is less frequent in *Exiguobacterium* genomes and was only found in strains S17 and SH31, showing an identity of 94%.

### Arsenic Resistance and Expression of Genes Required for Arsenic Detoxification

**FIGURE 4:**
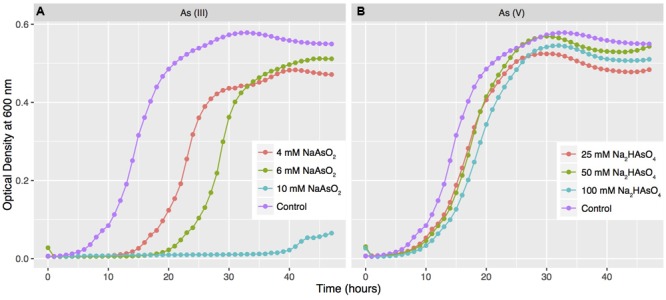
**Arsenic resistance of strain SH31.** Strain SH31 was grown in LB medium supplemented with different concentrations of As (III) **(A)** and As (V) **(B)**. OD_600_ readings were recorded every hour during 48 h. The results represent the mean of three experiments. Growth in media without arsenic was used as a control.

To determine if the presence of genes required for arsenic detoxification in strain SH31 correlated with increased arsenic resistance, we conducted growth curves with different As (III) and As (V) concentrations (**Figure 4**) and determined the MIC for each compound. Strain SH31 was able to grow in media containing up to 10 mM As (III) and 100 mM As(V), indicating that it is resistant to arsenic. A similar result was observed for strain S17.

The high arsenic resistance of strain S17 has been explained in part by the presence of the *acr3* gene (Ordoñez et al., 2015). A structural analysis showed that Acr3 from strains SH31 and S17 have an amino acid sequence identity of 94% and that key amino acids are conserved between both proteins. For example, residues Arg264, Asp109, and Glu294 are part of the core transmembrane segment and together with Cys107 could be required for As (III) binding. However, some variations were observed at different sections of the protein, (locus_02597-SP17) i.e., residues H92Y, R187Q, A311D, S34V, and S164A. We speculate that S34V, and S164A could be regulated by serine kinases (Supplementary Figure S2).

**FIGURE 5:**
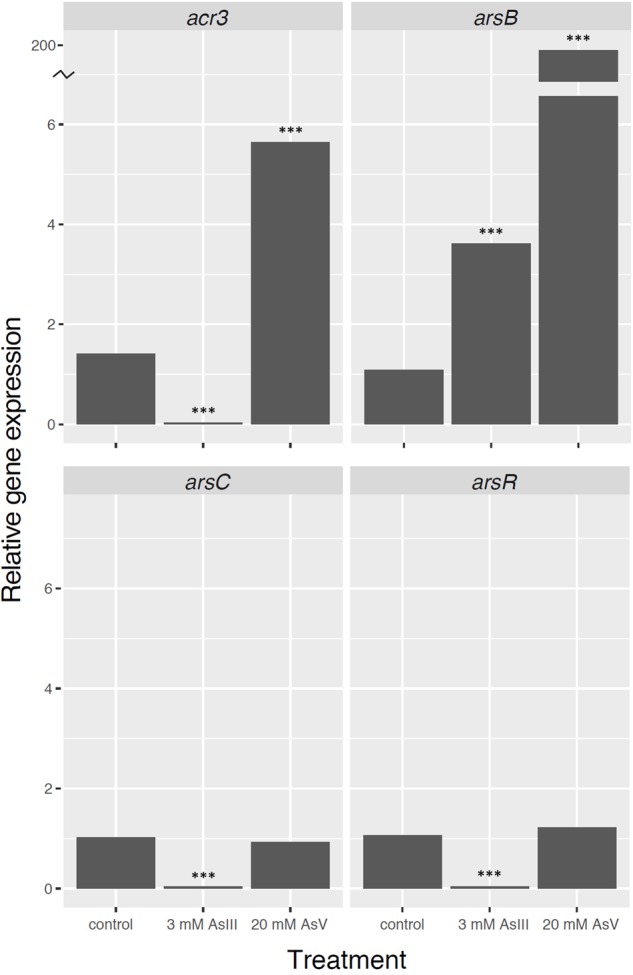
**Expression of genes required for arsenic resistance in *Exiguobacterium* sp. SH31 in response to As(III) and As(V) exposure.** Expression of the 16S rRNA gene was used as a normalizer. ^∗∗∗^*p* < 0.001.

To gain insights into the mechanism of arsenic resistance, strain SH31 was grown to mid-exponential growth phase, challenged with 3 mM As (III) or 20 mM As (V), and the transcript levels of genes required for arsenic resistance were quantified by qRT-PCR (**Figure 5**) and compared to the levels in untreated cells (control). The expression of *arsB*, encoding a membrane efflux transporter, increased in the presence of both As (III) and As (V), although in the later case a greater increase was observed (200 fold). Previous studies found a similar trend and showed that As (V) exposure resulted in higher transcript and protein levels of ArsB than with As (III) (Cleiss-Arnold et al., 2010; Belfiore et al., 2013; Andres and Bertin, 2016). No significant differences in the transcript levels of *arsC*, encoding arsenate reductase, were observed after As (V) exposure. Conversely, after As (III) exposure, *arsC* expression was strongly repressed. Previous studies reported that *arsC* is part of the *ars* operon in most bacteria (Páez-Espino et al., 2009; Kruger et al., 2013; Andres and Bertin, 2016); however, in the *Exiguobacterium* genus, *arsC* is not within the context of an operon (**Figure 3**). The expression of *arsR*, encoding the negative regulator of the *ars* operon, increased after As (III) exposure. As ArsR directly detects As (III) (Slyemi and Bonnefoy, 2012), this suggests that in strain SH31 As (III) exposure results in increased expression of the *ars* operon by decreasing the levels of ArsR.

The expression of *acr3* increased after As (V), but not As (III) exposure, in strain SH31. Interestingly, it was proposed that *acr3* expression is constitutive in strain S17 and does not change in response to arsenic (Ordoñez et al., 2015). This suggests that the expression of genes required for arsenic resistance could be different in both strains, although we cannot rule out that the observed result is caused by different experimental conditions between the studies.

## Discussion

The Chilean Altiplano harbors a series of ‘extreme’ environmental conditions that configures a specific and unique microbiota (e.g., Dorador et al., 2013; Albarracín et al., 2016). *Exiguobacterium* is a frequently isolated from different environments including other saline wetlands (e.g., Cuatro Ciénagas: Rebollar et al., 2012), where it has been determined that genetic differentiation of *Exiguobacterium* are defined by the ecology of diverse habitats.

Members of the *Exiguobacterium* genus carry a number of stress-related genetic determinants, including metal/metalloid resistance genes, which largely correlate with the phylogenetic structure (**Figure 2**). In particular, *Exiguobacterium* SH31 (Group II) encodes proteins required for arsenic resistance, including Acr3, which has only been described in *Exiguobacterium* S17 (Ordoñez et al., 2015) (**Figures 3**, **5**). We also found that the accessory genome of *Exiguobacterium* is large, and that the presence of genes involved in interactions with the environment (e.g., catalytic activity, binding, metabolic process) is variable (Supplementary Figure S1). Altogether, these results suggest that the *Exiguobacterium* genus is cosmopolitan, diverse, and possibly ancient, with a broad repertoire of genetic elements that allow them to efficiently adapt to local environmental conditions (**Figure 2**).

*Exiguobacterium* strains showed variability in the GO categories related to adaptation, such as catalytic activity, transporter activity, nucleic acid binding, transcription factor activity, metabolic process, etc. Conversely, less variability was observed in the GO categories related to fundamental cellular functions, e.g., structural molecule activity, membrane constituents, macromolecular complex, cell parts, and biological regulation. In agreement with our findings, Yumoto et al. (2004) reported that *E. oxidotolerans* JCM 12280 differed from other closely related strains in their physiological and biochemical characteristics, e.g., growth temperature range, acid production from several substrates and enzymatic activities. Our analyses support these results, showing that *E. oxidotolerans* JCM 12280 appears to be distinct based on its phylogenetic position, ANI similarity, and stress-response gene repertoire.

In agreement with the high levels of radiation that it must withstand in its environment (Hernández et al., 2016; Molina et al., 2016), the genome of strain SH31 comprises several genes encoding proteins required for UV radiation resistance, including the *uvr, mut, rec* and *ruv* systems; the *ssb, lexA, phrB* and *cry-DASH* genes, photoprotective pigments and those required for detoxifying oxidative stress (Kurth et al., 2015). Surprisingly, strain SH31 did not show any highly relevant distinctive features. For example, previous studies found four copies of *ssb* in the genome of strain S17 (Ordoñez et al., 2013, 2015). In *Deinococcus radiodurans*, the presence of high copy numbers of *ssb* was proposed to be responsible for its superior radioresistance and DNA repair efficiency (Eggington et al., 2004; Cox and Battista, 2005; Norais et al., 2009). Long read sequencing technologies such as PacBio or Oxford Nanopores could aid in determining with certainty whether SH31 has experienced the duplication of gene families involved in stress response.

Some *Exiguobacterium* strains are resistant to NaCl and heavy metals such as Pb, Cu, and Hg (Petrova et al., 2002; Bian et al., 2011). In particular, *Exiguobacterium* isolated from permafrost sediments in Russia and Canada that contained 0.001 – 2.9% of mercury carried the *mer* operon (Petrova et al., 2002). Our results show that all of the *Exiguobacterium* strains included in the analyses have the *merA* gene, but that only 6 strains had additional genes of the operon (Supplementary Table S4). The *mer* operon can be located in the chromosome, plasmids, or transposons, and has been studied as a mechanism of horizontal dissemination that confers a broad spectrum of mercury resistance among *Bacillus* and related species (Bogdanova et al., 2001). Other strains such as ZM-2, isolated from agricultural soils irrigated with tannery effluents in India, resist up to 12.4 mM of potassium chromate (Alam and Malik, 2008), and some isolates tolerate up to 1.5 mM and reduce 0.75 mM of Cr(VI) (Sarangi and Krishnan, 2008). Additionally, some *Exiguobacterium* isolates are able to reduce Cr(VI) anaerobically in a large range of temperatures, pH and salt concentrations (Pattanapipitpaisal et al., 2002; Okeke, 2008). Our comparative genomics approach confirms that all 34 *Exiguobacterium* strains possess the genes encoding proteins required for chromate reduction and efflux, namely *chrR* and *srpC*, respectively, which could be responsible for chromate resistance in this genus.

*Exiguobacterium* sp. SH31 is more closely related to the arsenic resistant strain S17 (Belfiore et al., 2013). The high arsenic resistance of strain S17 has been explained by an increased number of genes encoding proteins required to detoxify this toxic compound and by the presence of the *acr3* gene, which confers increased arsenite and arsenate resistance (10 and 150 mM, respectively; Belfiore et al., 2013; Ordoñez et al., 2015). In agreement, strain SH31 is also arsenic resistant, which could be explained by the presence of several proteins required for its detoxification, including Acr3.

In the post-genomic era, high-throughput technologies have enabled researchers with the power to interrogate genomes from the dark matter of the microbial tree of life (Wu et al., 2009). Yet, the traditional use of morphology, phenotype, biochemical traits, and single-gene inferences to classify microorganisms implies that our current understanding of what constitutes a species or genus is imperfect (Hugenholtz et al., 2016). Herein, we show that using an extreme cut-off of 75% ANI in a relatively small dataset yielded as many as six clusters in an otherwise unified genus. While comparative genomics studies of polyextremohiles are needed to understand their distribution, evolutionary history, and biotechnological potential, thorough sampling designs, metagenomics-based studies, and functional assays such as those based on metabolomics, transcriptomics, and proteomics, will enable researchers to develop a systems level understanding of the patterns and processes leading to molecular adaptation.

## Author Contributions

JF, PA, CD, and FR performed field work, processed samples and sequenced data; JC-S, EC-N, and CPS conceived and designed the study; JC-S, FR, and CS performed the experiments; JC-S, SV, DA, and EC-N analyzed data; RQ, FM, EC-N, CD, FR, and CPS contributed with reagents/materials/analysis tools; JC-S, CP-E, EC-N, FR, and CPS wrote the paper. All authors read and approved the final manuscript.

## Conflict of Interest Statement

The authors declare that the research was conducted in the absence of any commercial or financial relationships that could be construed as a potential conflict of interest.

## Footnotes

1 www.dsmz.de

2 http://bioinf.comav.upv.es/seq_crumbs/index.html

3 https://github.com/microgenomics/plotMyGBK

4 http://bioconductor.org/packages/release/bioc/html/OmicCircos.html

5 https://github.com/widdowquinn/pyani
