# Supplementary material for: Comparative Genomics Analysis of a New Exiguobacterium Strain from Salar de Huasco Reveals a Repertoire of Stress-Related Genes and Arsenic Resistance
Source: Front Microbiol. 2017 Mar 21;8:456. doi: 10.3389/fmicb.2017.00456 (PMC5360010; doi:10.3389/fmicb.2017.00456)
Supplement: Supplementary file 5 [file Image_1.PDF]

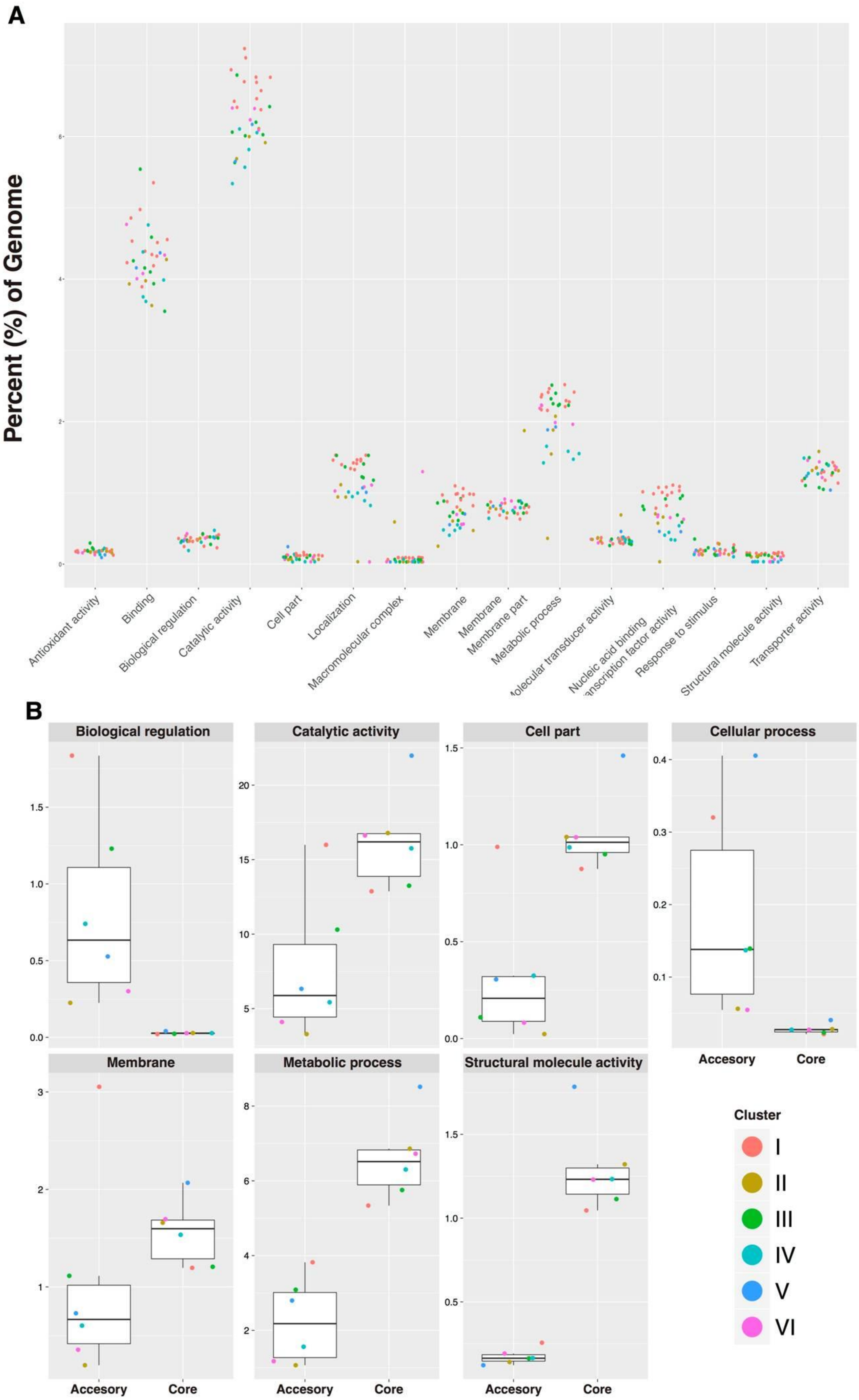

Supplementary figure S1. Functional analysis of core and accessory genes. **(A)** distribution of GO functional terms among all the strains accessory genome as a variability measure. **(B)** Box-and-whisker plots of functional terms overrepresented in core genes compared to accessory genes among genomic clusters. The box plots were drawn with the upper and lower lines corresponding to the first and third quartiles, the middle lines corresponding to the medians, and the upper and lower whiskers extended to 1.5 times the interquartile ranges. Color dots represent each cluster value. Note the difference in scale between the panels.
