# Supplementary material for: Comparative Genomics Analysis of a New Exiguobacterium Strain from Salar de Huasco Reveals a Repertoire of Stress-Related Genes and Arsenic Resistance
Source: Front Microbiol. 2017 Mar 21;8:456. doi: 10.3389/fmicb.2017.00456 (PMC5360010; doi:10.3389/fmicb.2017.00456)
Supplement: Supplementary file 6 [file Image_2.PDF]

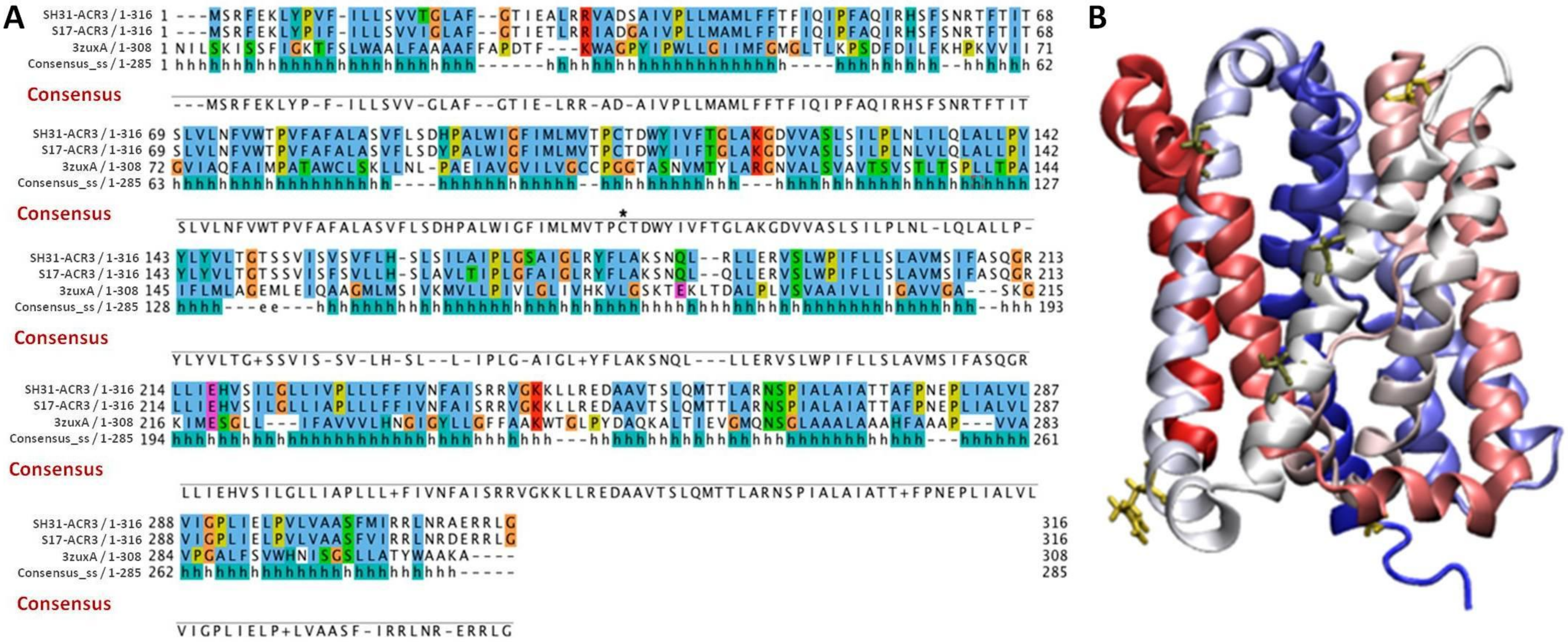

Supplementary figure S2. a) SH31-ACR3, S17-ACR3 and 3zuxA sequence alignment and Secondary structure of 3zuxA template. B) Homology model of Acr3 based on the Sodium Bile transporter (pdb:3zuxA). Acr3 amino-acid variations are highlighted.
